# Supplementary material for: Field-free spin-orbit switching of perpendicular magnetization enabled by dislocation-induced in-plane symmetry breaking
Source: Nat Commun. 2023 Sep 6;14:5458. doi: 10.1038/s41467-023-41163-3 (PMC10482861; doi:10.1038/s41467-023-41163-3)
Supplement: Supplementary file 1 — Supplementary Information [file 41467_2023_41163_MOESM1_ESM.pdf]

Supplementary materials for

Field-free Spin-orbit Switching of Perpendicular Magnetization Enabled by Dislocation-induced In-plane Symmetry Breaking

Yuhan Liang[1,†], Di Yi[1,†], Tianxiang Nan[2,†], Shengsheng Liu[1,3] , Le Zhao[4,5], Yujun Zhang[6], Hetian Chen[1], Teng Xu[4,5], Minyi Dai[7], Jia-Mian Hu[7], Ben Xu[8], Ji Shi[9], Wanjun Jiang[4,5,*], Rong Yu[1,3, *], Yuan-Hua Lin[1,*]

1 *School of Materials Science and Engineering, Tsinghua University, Beijing 100084, China,*

2 *School of Integrated Circuits and Beijing National Research Center for Information Science and Technology (BNRist), Tsinghua University, Beijing 100084, China*

3 *National Center for Electron Microscopy in Beijing, Tsinghua University, Beijing 100084, China*

4 *State Key Laboratory of Low-Dimensional Quantum Physics and Department of Physics, Tsinghua University, Beijing 100084, China*

5 *Frontier Science Center for Quantum Information, Tsinghua University, Beijing 100084, China*

6 *Institute of High Energy Physics, Chinese Academy of Sciences, Beijing 100049, China*

7 *Department of Materials Science and Engineering, University of Wisconsin–Madison, Madison, WI 53705, USA.*

8 *Graduate School, China Academy of Engineering Physics, Beijing 100193, China*

9 *School of Materials and Chemical Technology, Tokyo Institute of Technology, Tokyo 152-8552, Japan*

* Corresponding author. Email: Jiang_lab@tsinghua.edu.cn (W.-J. J.); ryu@tsinghua.edu.cn (Y.R.); linyh@tsinghua.edu.cn (Y.-H. L.)

† These authors contribute equally to this work

Note 1: The theoretical calculation of tilting of crystal lattice induced by out-of-plane Burgers vector component

Considering the small tilting angle of metal layers, to calculate the tilting angle θ_e, we use the equation $\theta_e = \frac{B}{a/\delta}$ that is used for the description of low-angle boundary, whereas δ is the lattice mismatch, a is the lattice constant of NiO, and B is the length of Burgers vector, see schematic in Supplementary Fig. 6.

Considering the case that Pt contacts with NiO:

$$\theta_e^{min}=\frac{B}{D}=\frac{B}{a/\delta}=\frac{\sqrt{2}}{4}\times\frac{4.17\text{-}3.92}{4.17}\text{rad} = 0.0212\text{ rad} = 1.214°$$

The dislocation density is calculated as $n_{dislocation} = 1/D_{Pt\text{-}NiO} = 1.44\times10^6$ /cm.

Considering the case that FCC Co contacts with NiO:

$$\theta_e^{max}=\frac{B}{D}=\frac{B}{a/\delta}=\frac{\sqrt{2}}{4}\times\frac{4.17\text{-}3.55}{4.17}\text{rad} = 0.0526\text{ rad} = 3.01°$$

The dislocation density is calculated as $n_{dislocation} = 1/D_{Co\text{-}NiO} = 3.57\times10^6$ /cm.

Here, D is the distance between two neighboring dislocations. We note that the calculated θ_e is the average value around the core of dislocation. Considering both the elastic stiffness and film thickness of Pt are much larger than those of Co[1], the Co layer would be fully strained by the Pt layer to form a coherent interface between Pt and Co. Therefore, the crystal tilting would be close to the case of Pt on NiO. The experimentally measured tilt angle is very close to above estimation. The experimentally measured dislocation density is about 4.11×10^6/cm for (100)-oriented heterostructures, and 3.70×10^6/cm for (110)-oriented heterostructures, respectively. These values are close to theoretical values, see Supplementary Fig. 5.

Note 2: The simulation of angle dependent R_{xy} via rotating external field

The single domain model is adapted for the calculation of polar angular dependent Hall resistance (R_{xy}) curve. For simplicity, here, we assume the energy of system can be expressed as:

$$E(\theta) = -K_u\cos^2(u - u_M) - \mu_0 H M_s \cos(u - \gamma)$$

where the first term describes the tilted anisotropy, and the second term represents the Zeeman energy. In this formula, u is the polar azimuth of the magnetic moment. u_M and γ represent the polar azimuths of the easy axis and applied magnetic field, respectively. We note that u_M = 90°-θ_M, where θ_M is the angle of tilted magnetic easy axis that is defined in main text. The geometry is shown in Supplementary Fig. 14. For each γ, we calculate the direction of magnetic moment by using the energy minimal E(u), and further get the M_z by using M_z=$M_s$sin (θ_M), as shown in Supplementary Fig. 14. Based on the simulation results, the angular dependence of Hall resistance in Fig. 2**d** reveals the presence of tilted magnetic easy axis with θ_M close to 20°. Similar results have also been reported in $SrRuO_3$ films[2].

Note 3: The simulation of tilted magnetic easy axis induced by the tilting of crystal lattice

According to previous studies on (110)-oriented Pt/Co heterostructures, the Co shows strong in-plane uniaxial anisotropy due to magneto-elastic effect, and the in-plane easy (hard) axis is along [001] ([$\bar{1}$10])-direction[3-5]. Thus, in our (110)-oriented Pt/Co/oxide heterostructures, we mainly consider the magneto-elastic energy (K_e), shape anisotropy ($-2\pi M_s^2$) and perpendicular magnetic anisotropy (K_{PMA}) originating from Pt/Co or Co/oxide interfaces. Because of the tilting of crystal lattice, the [001] direction of Pt/Co is slightly tilted by θ_e as shown in Figs. 1**f** and 2**d**. Given that the K_e is strongly coupled to lattice due to spin-orbit coupling, it is expected that the angular dependence of K_e is also affected by θ_e. Thus, the energy of the (110)-oriented Pt/Co/oxide heterostructures in ($\bar{1}$10) plane can be written as:

$$E = -K_{PMA}\sin^2(\theta)+2\pi M_s^2\sin^2(\theta)+K_e\sin^2(\theta-\theta_e) = -K_{eff}\sin^2(\theta) + K_e\sin^2(\theta-\theta_e)$$

Here, $K_{eff} = K_{PMA} - 2\pi M_s^2$ is the effective K_{PMA}, θ is the direction of magnetization and θ_e is the tilting angle of crystal lattice, as schematically shown in Supplementary Fig. 16**a**.

Take $\xi = \frac{K_{eff}}{K_e}$, the tilted angle θ_M of magnetic easy axis is determined as the energy minimum of E(θ), which is found to depend on both ξ and θ_e. As shown in Supplementary Fig. 16**b**, for $\theta_e = 0°$, i.e. no tilting of crystal lattice, only in-plane easy axis ($\theta_M = 0°$) and perpendicular easy axis (θ_M= 90°) are allowed. By contrast, for $\theta_e \neq 0°$, the tilted magnetic easy axis can be induced, and

θ_M increases with increasing ξ. Representatively, the angular dependences of anisotropy energy ($E(\theta)$) for ξ = 0.8, 0.9, 1.2 with a fixed $\theta_e = 1.2°$ are shown in Supplementary Fig. 16**c**, confirming the tilted magnetic easy axis.

According to the previous studies, the in-plane anisotropy K_e caused by magneto-elastic effect in (110)-oriented Pt/Co heterostructure is estimated to be about 1.5×10^7 erg/cm^3 ~ 5.3×10^7 erg/cm^3 (1.5 MJ/m^3 ~ 5.3 MJ/m^3)[1], and the effective perpendicular magnetic anisotropy K_{eff} for t_{Co} = 0.6 nm is estimated to be about 5.4 MJ/m^3 [6]. Thus, the magnitudes of K_{eff} and K_e are comparable in our heterostructures, consistent with the criteria that favor the titled magnetic easy axis as shown in Supplementary Fig. 16**b**.

Note 4: The estimation of spin-torque efficiency measured by second Harmonic Hall (SHH) resistance

Here, we utilized second harmonic Hall (SHH) resistance to measure the spin-torque efficiency induced by SOT. The geometry set-up for the measurement is shown in Supplementary Fig. 20**a**, the ac current $I = I_0\sin(\omega t)$ (frequency $f = \omega/2\pi$ = 233.33 Hz) is applied along x-direction (x-direction along [$\bar{1}$10] or [001]). Two lock-in amplifiers are utilized to record the first ($V_{1\omega}$) and second ($V_{2\omega}$) harmonic Hall voltage during sweeping in-plane magnetic field H_x. The first harmonic Hall voltage $V_{xy}^{1\omega}$ mainly corresponds to the direction of magnetization. The second harmonic Hall voltage $V_{xy}^{2\omega}$ is correlated to the oscillation of magnetization around the equilibrium position, which is induced by SOT-generated effective field.

As the external magnetic field H_x larger than anisotropic field H_K, the magnetization vector **M** is mainly along the x-direction, leading to the SHH resistance written as[7, 8]:

$$R_{xy}^{2\omega}=\frac{R_{AHE}}{2}\frac{H_{DL}}{|H_x|-H_K}+R_{PHE}\frac{H_{FL}}{|H_x|}+R_{thermal}$$

Where R_{AHE} and R_{PHE} are the AHE and planar Hall resistances and $R_{thermal}$ is the thermal contribution from anomalous Nernst and spin Seebeck effect. Since $R_{PHE} \ll R_{AHE}$, the second term can be neglected. Thus, we can estimate the effective field H_{DL} via fitting the $R_{xy}^{2\omega}$ data in the large in-plane field regime.

We then apply ac current along [001] and [$\bar{1}$10] directions with in-plane field parallel to the

current in sample Pt(5)/Co(0.6)/Pt(1.4)/NiO(20)/MgO(110), the results are shown in Supplementary Fig. 20**b** and 20**c**. By changing the amplitude of ac current and fitting the SHH resistance, as shown in Supplementary Fig. 20**d**, we estimate the H_{DL}/J_e along [001] is about 4.25 mT per 10^{11} A/m^2, and the H_{DL}/J_e along [$\bar{1}$10] is about 4.83 mT per 10^{11}A/m^2. These values are close to the recorded value in similar Pt/Co heterostructures[9]. In addition, the damping-like spin-torque efficiency can be estimated as $\xi_{DL}=\frac{2e}{\hbar}M_s t_{Co}\frac{H_{DL}}{J_e}$. Here, we take the saturated magnetization of Co as M_s = 1000 emu/cm^3, and t_{Co}= 0.6 nm for the thickness of Co. By taking the H_{DL}/J_e of two directions, the damping-like torque efficiency ξ_{DL} along [$\bar{1}$10] ($\xi_{DL}^{[\bar{1}10]}$) is calculated as 0.088, and ξ_{DL} along [001] ($\xi_{DL}^{[001]}$) is about 0.078, respectively.

We also apply transverse field in SHH measurement, e.g. current along [$\bar{1}$10] ([001]) as field is along [001] ($\bar{1}$10), which can characterize field-like effective field[10]. However, the $R_{xy}^{2\omega}$ measured by transverse magnetic field is about 1 or 2 order lower than the $R_{xy}^{2\omega}$ measured by longitude magnetic field (see the insets of Supplementary Fig. 20**b** and Fig. 20**c**). Thus, the field-like torque can be neglected, and the damping-like torque plays the main role in SOT switching, which is consistent with previous studies[8,11].

Note 5: The heating effect induced by current pulse

The device temperature could be raised up during the application of current pulse due to the Joule heating effect ($\propto I^2R$). Here, we check the temperature increase of the representative devices, see Supplementary Fig.21. We first measure the longitude resistance (R_{xx}) in devices with θ_M = 16° and θ_M = 84° devices, which is done by using a small dc current of 0.1 mA, see Supplementary Fig.21**a**. The dc current of 0.1 mA is too small to induce an appreciable Joule heating. Then we measure the R_{xx} as a function of the amplitude of the *ac* current density (R_{xx} ~ J), see Supplementary Fig.21**b**. Thus, the temperature raises during SOT switching can be estimated by comparing the temperature dependence of resistance with the current-induced resistance changes. The current-induced temperature increase (ΔT) in SOT switching process is estimated to be up to 95 K for θ_M = 16° device and 80 K for θ_M = 84° device. Note that the temperature rising should be overestimated, considering that the pulse width for SOT switching is only 2 ms. In addition, we measure the temperature dependent AHE of aforementioned devices (300 K to 380 K), see Supplementary

Fig.21**c**. With the temperature increasing, the magnetization slightly tilts towards the film plane and coercivity decreases, which could be beneficial for the field-free SOT switching. Thus, the Joule heating plays a partial role in assisting the field-free SOT switching, in the presence of the dislocation-induced in-plane symmetry breaking.

Note 6: Field-dependent SOT switching results

The schematic of field-dependent SOT switching measurements is shown in Supplementary Fig. 24, in which the external magnetic field is applied parallel to current pulse. For Pt(5)/Co(1.2)/NiO(20)/MgO(110) (θ_M = 16°), as the current pulse is applied along $[\bar{1}10]$, the magnetic field up to 100 mT cannot suppress the SOT switching, see Supplementary Fig. 24**a**. However, as the current pulse is applied along [001], the current switching is prohibited regardless the direction of in-plane fields, as shown in Supplementary Fig. 24**b**. For Pt(5)/Co(0.6)/Pt(1.4)/NiO(20)/MgO(110) (θ_M = 84°), as the current pulse is applied along $[\bar{1}10]$, the external magnetic field about 2.2 mT can compensate the SOT switching, see Supplementary Fig. 22**c** below. As the current pulse is applied along [001], the SOT switching can be induced by in-plane magnetic field, see Supplementary Fig. 24**d**.

The distinct field-dependent SOT switching behavior of two heterostructures can be understood by considering the combination effects of the torque $\vec{\tau}_{ext}$ induced by external field $\vec{H}_{ext}$, SOT and the orientation of the easy axis (titled magnetic anisotropy). When the current pulse is applied along $[\bar{1}10]$, the damping-like torque forces the magnetization to align parallel to $\vec{\boldsymbol{\sigma}}$ (along [001] as shown in Supplementary Fig. 25**a** and Fig. 25**c**)[12]. If the $\vec{H}_{ext}$ is applied parallel to the current pulse, the $\vec{\boldsymbol{\tau}}_{ext} \propto \vec{\mathbf{M}} \times \vec{\mathbf{H}}_{ext}$ will force the magnetization tilt towards the hard axis. For θ_M = 16°, the hard axis is far away from the sample-plane and the $\vec{\tau}_{ext}$ cannot align the magnetization towards the hard axis, thus the $\vec{\mathbf{H}}_{ext}$ up to 100 mT could not compensate the titled magnetic anisotropy, leading to a robust SOT switching behaviors, see Supplementary Fig. 25**a** below. For θ_M = 84°, the hard axis is close to the sample-plane (6°), thus the $\vec{H}_{ext}$ of 2.2 mT could compensate the titled anisotropy, leading to the change of SOT switching behaviors, see Supplementary Fig. 25**c**.

When the current pulse is applied along [001], the damping-like torque forces the magnetization to align parallel to $\vec{\boldsymbol{\sigma}}$ (along $[\bar{1}10]$ as shown in Supplementary Fig. 25**b** and Fig. 25**d**).

It is noted that $[\bar{1}10]$ is the magnetic hard axis (see Fig. 2 of revised Manuscript) and the tilted easy axis is within the plane perpendicular to $[\bar{1}10]$. Therefore, deterministic SOT switching of perpendicular magnetization is not expected without $\vec{\mathbf{H}}_{\mathrm{ext}}$. For θ_M = 16°, the magnetic easy axis is very close to the [001]-direction. Thus the $\vec{\mathbf{H}}_{\mathrm{ext}}$ along [001] would strongly favor magnetization along this direction, resulting in the absence of SOT switching, see Supplementary Fig. 25**b**. For θ_M = 84°, the easy axis is close to out-of-plane [110]-direction and the application of $\vec{H}_{ext}$ would assist SOT switching (see Supplementary Fig. 25**d**).

Note 7: The comparison with other studies based on tilted magnetic anisotropy.

Previous studies have also revealed other methods to induce titled anisotropy and field-free SOT switching. Here, we will compare our strategy with these methods. Firstly, as shown in reference 15, the tilted anisotropy is induced by fabricating a wedge-like nanomagnet device with lateral size of a few hundred nanometers, which requires complex nano-fabrication process. Secondly, as shown in reference 16, the tilted magnetic anisotropy can be induced by interlayer magnetic coupling in the multilayer structures, which demands a very precise control of thickness in each layer. In addition, the additional layers also impair the inherent advantages of a single layer magnet. Thirdly, as shown in reference 17, an easy cone anisotropy is induced, and the deterministic SOT switching is induced by the remnant in-plane magnetization component. However, due to the special type of anisotropy, the zero-field SOT switching ratio determined by ΔR_{xy} is very small (less than 2%). Furthermore, none of these studies have carefully checked the endurance of SOT switching.

By contrast, in our proposed method by designing crystal symmetry and dislocation, we can realize the tilted magnetic anisotropy in the single-layer ferromagnetic film, without the use of complex fabrication or interlayer coupling. The field-free SOT switching is achieved with relatively low critical current density (10^{11}A/cm^2), high switching ratio (70%), and good endurance (>10^4 cycles). These highlight the advantages of our method that could be beneficial for future applications. The important parameters are tabulated in Supplementary Table 1.

Supplementary Figures

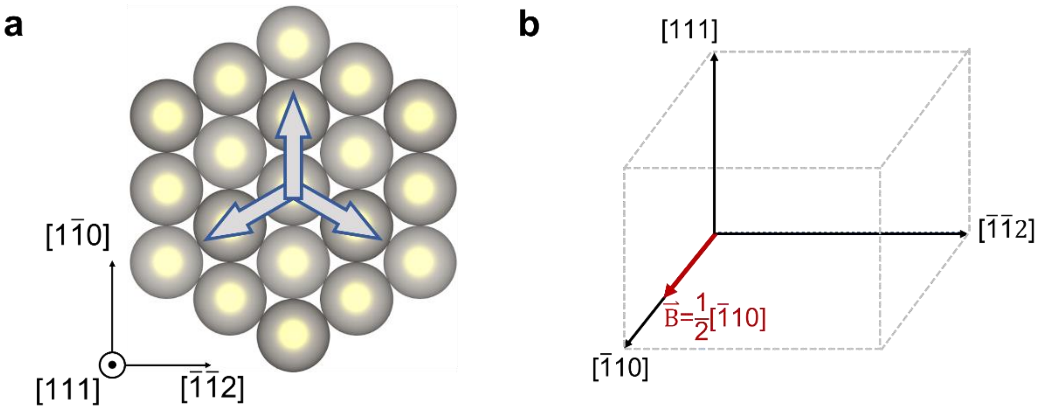

Supplementary Figure 1. The additional analysis of Burgers vectors in heterostructure with (111)-orientation. a, For the (111)-orientation, there are three equivalent $\frac{1}{2}$<110> lattice vectors in the film plane, resulting in **b,** dislocation with only in-plane Burgers vectors and no tilting of crystal lattice.

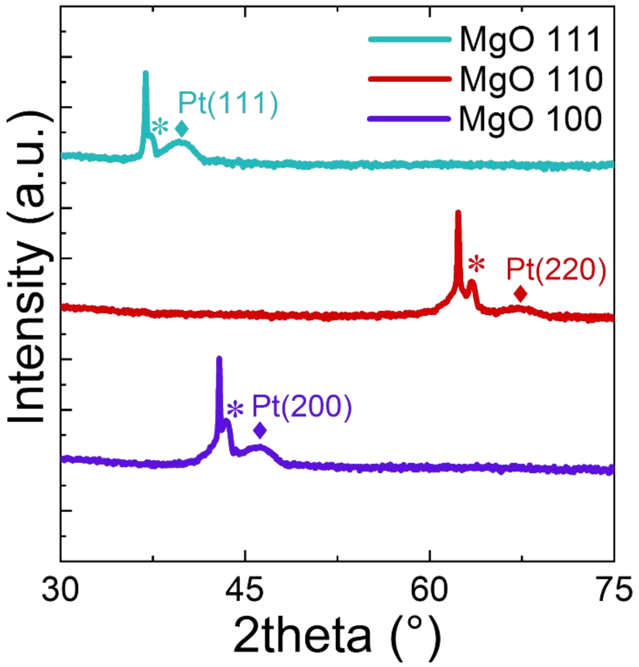

Supplementary Figure 2. The XRD results. The XRD patterns for Pt(5)/Co(1.5)/NiO(20)/MgO heterostructures with (100)-, (110)- and (111)-orientation are displayed, respectively. The NiO peaks are noted by symbol *. The highest peaks correspond to MgO substrates.

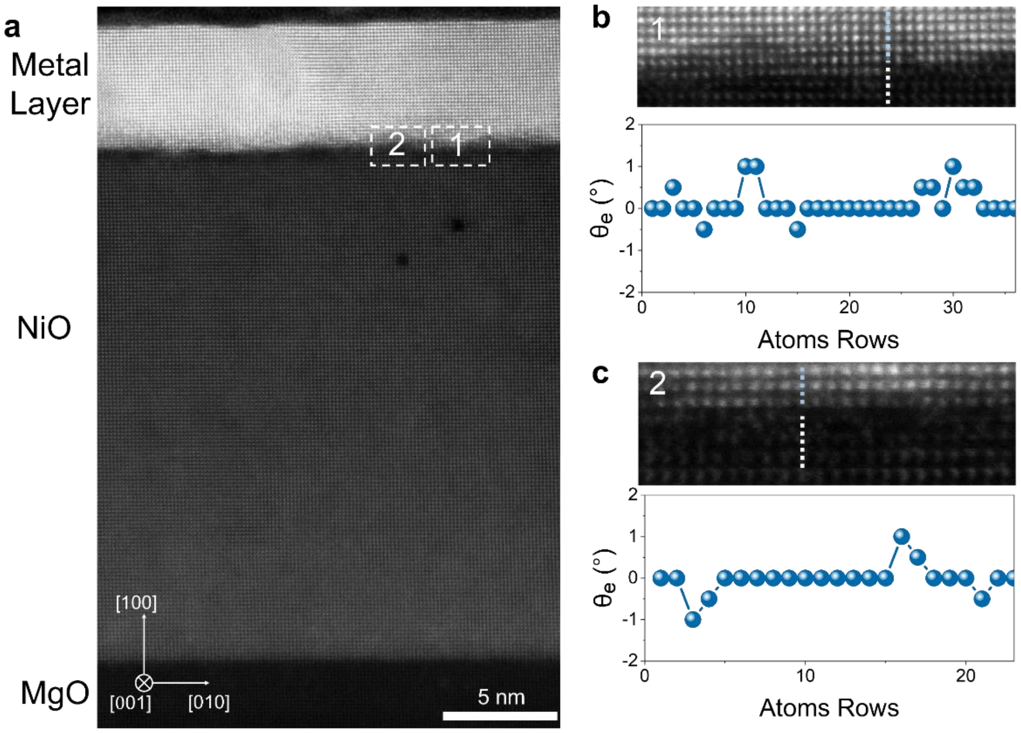

Supplementary Figure 3. The cross-section HAADF-STEM images of the (100)-oriented Pt(5)/Co(1.5)/NiO(20)/MgO. a, the large-scale HAADF-STEM image. Lateral statistic results of θ_e of selected areas 1 and 2 are shown in **b** and **c**, respectively. The areas are denoted by white dash boxes in **a**. The blue and white dash lines represent the lattice of metal layer and NiO.

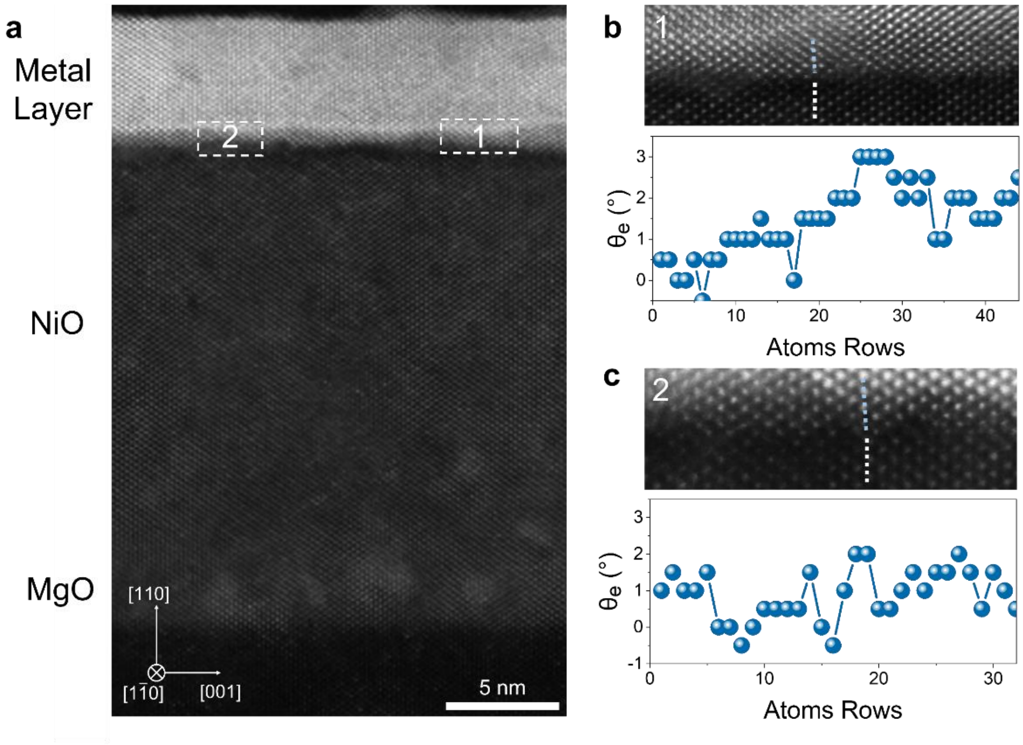

Supplementary Figure 4. The cross-section HAADF-STEM images of the (110)-oriented Pt(5)/Co(1.5)/NiO(20)/MgO. a, the large-scale HAADF-STEM image. Lateral statistic results of θ_e of selected areas 1 and 2 are shown in **b** and **c**, respectively. The areas are denoted by white dash boxes in **a**. The blue and white dash lines represent the lattice of metal layer and NiO.

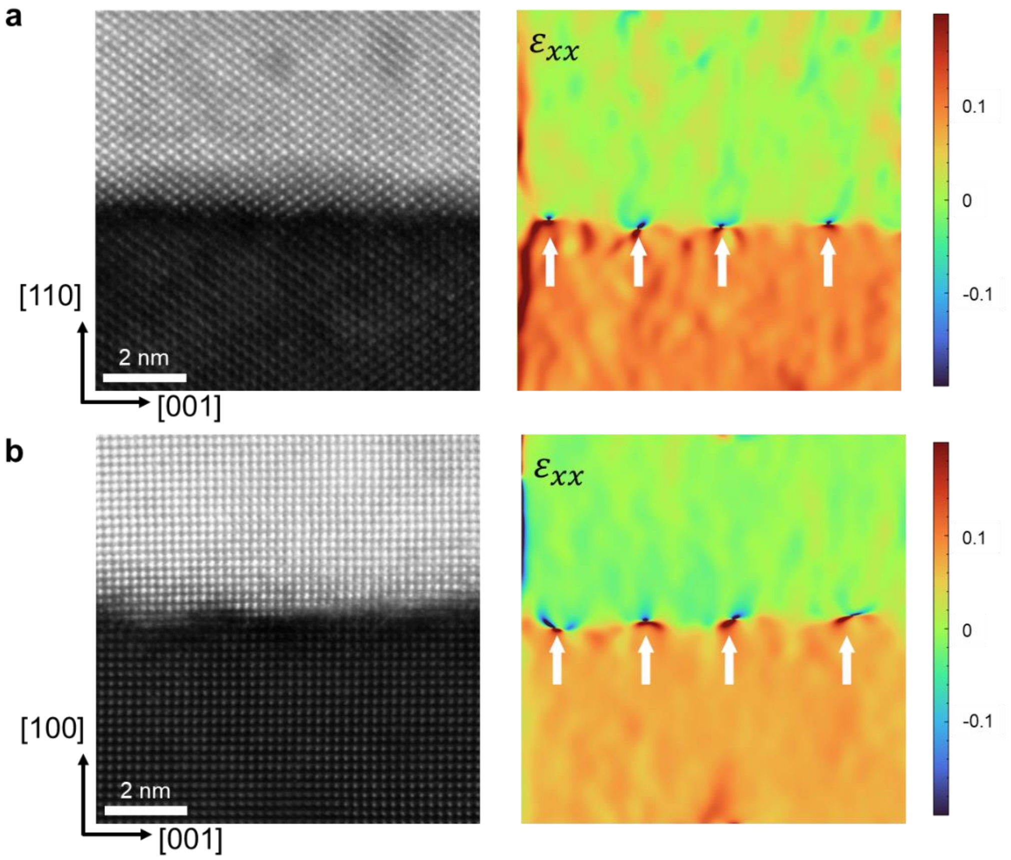

Supplementary Figure 5. The dislocation arrays confirmed by geometry phase analysis (GPA). a, the HADDF image and GPA analysis result of Pt(5)/Co(1.5)/NiO(20)/MgO(110). **b,** the HADDF image and GPA analysis result of Pt(5)/Co(1.5)/NiO(20)/MgO(100). The dislocations are denoted by white arrows.

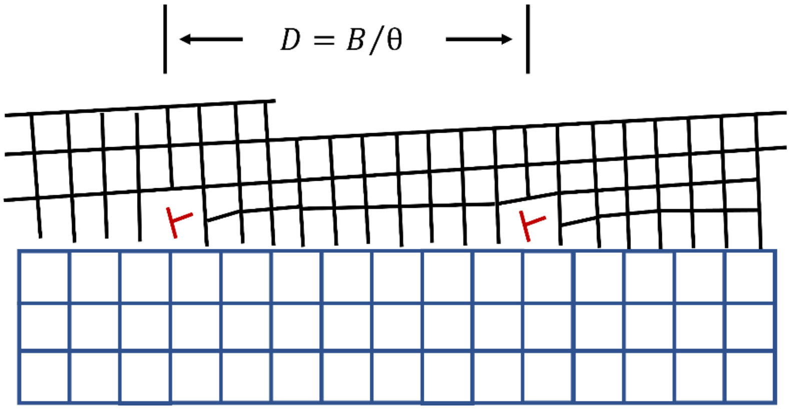

Supplementary Figure 6. The schematic diagrams for crystallographic tilting induced by dislocations. The calculation of crystallographic tilting angle θ_e is carried out by using the low-angle boundary scenario, see Supplementary Note 1.

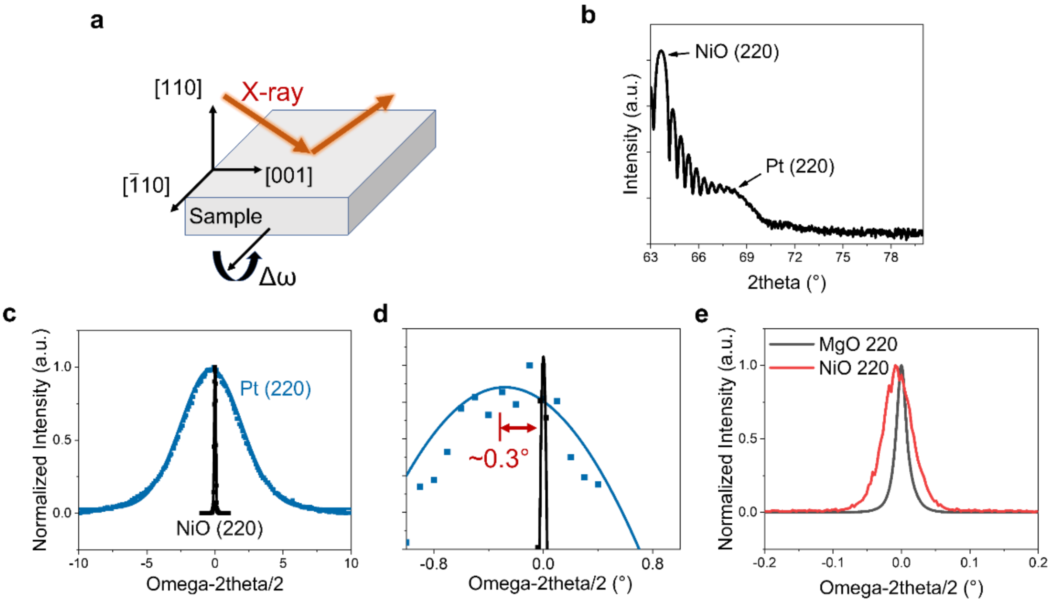

Supplementary Figure 7. Tilting of crystal lattice of Pt detected by XRD. The synchrotron-based X-ray diffraction (XRD) for rocking curve is carried out in 1W1A, Beijing Synchrotron Radiation Faculty, Beijing, China. The wavelength of X-ray is 1.5438 Å, and angular resolution is 0.042°. The laboratory XRD is carried out by high-resolution X-ray direction equipment, Empyrean, Malvern Panalytical, with the conventional X-ray of Cu $K_{\alpha 1}$. **a-d,** the synchrotron based XRD results. **a,** the geometry set of the rocking curve measurement. **b,** the theta-2theta scanning of the (110)-oriented Pt(5)/Co(1.5)/NiO(20)/MgO between 63° and 80°. **c,** the rocking curves of NiO (220) peak and Pt (220) peak. **d,** enlarged area around peaks shows the Pt layer is titled by about 0.3° with respect to NiO (average $\theta_e \sim 0.3°$). **e,** the rocking curve of MgO and NiO (220) peak by laboratory XRD, showing negligible tilting of NiO with respect to MgO substrate (~0.01°).

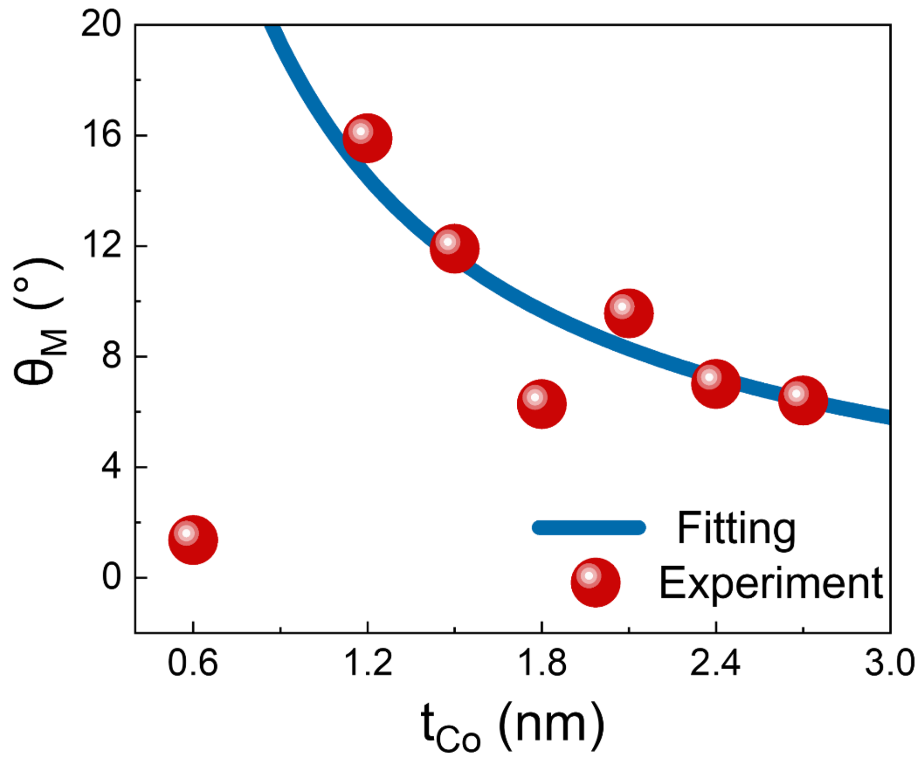

Supplementary Figure 8. The dependence of tilted angle θ_M on thickness of Co (t_{Co}). The estimated θ_M with various t_{Co} is determined by the field dependent R_{xy} of the (110)-oriented Pt(5)/Co(t_{Co})/NiO(20)/MgO heterostructures with various t_{Co}. The blue line is the $1/t_{Co}$ fitting of the experimental data.

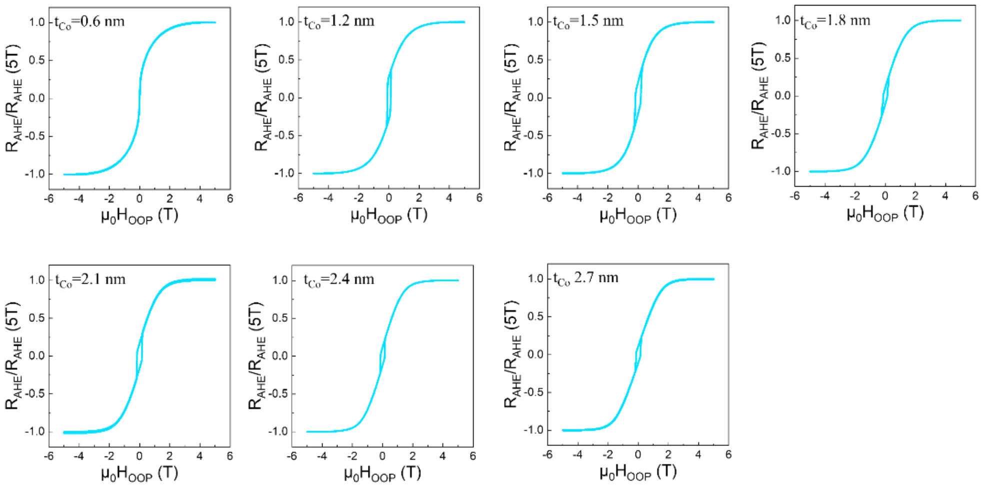

Supplementary Figure 9. The normalized AHE signal of (110)-oriented Pt(5)/Co(t_{Co})/NiO(20)/MgO heterostructures with various t_{Co}. The tilted angle θ_M is calculated by θ_M=arcsin [R_{xy}(H=0)/R_{xy}(H = 5T)].

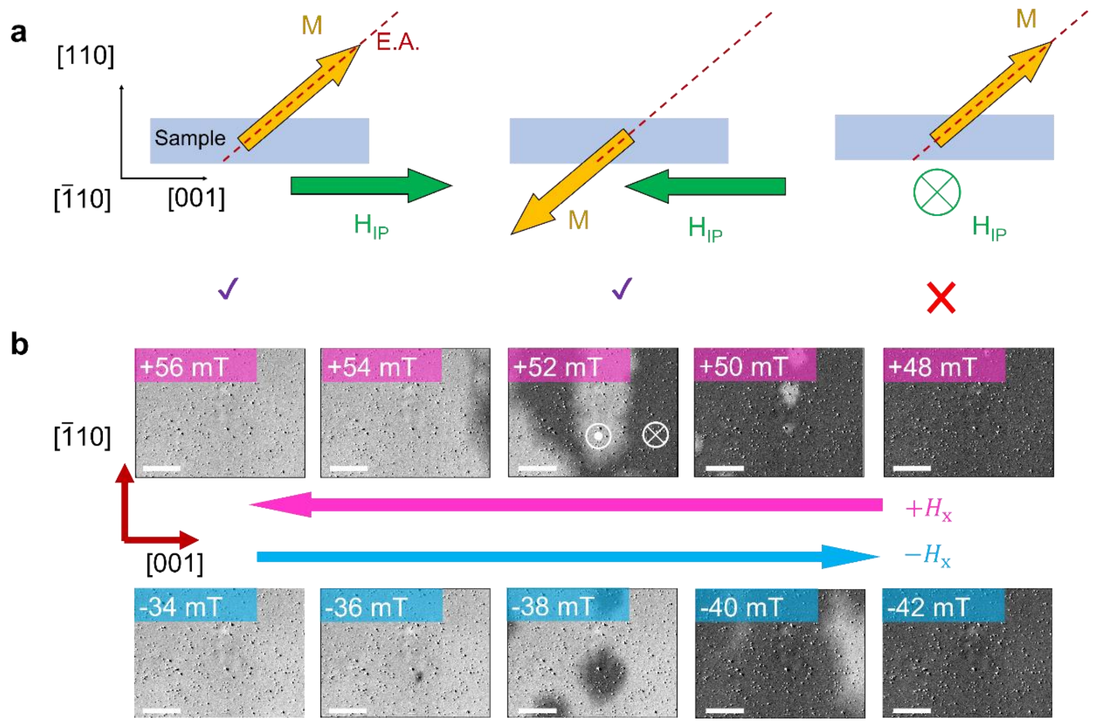

Supplementary Figure 10. Switching of perpendicular magnetization component by in-plane magnetic field. a, The schematic diagrams of the switching of perpendicular magnetization by in-plane field H_{IP}. Because the tilted magnetic easy axis lies in the first and third quadrants defined by [110] and [001], the symmetry between magnetization and in-plane magnetic field H_{IP} is broken. For H_{IP} along [001] ([00$\bar{1}$]), the upward (downward) state is favored. For H_{IP} along [$\bar{1}$10], two states are equivalent, and the perpendicular magnetization component cannot be switched by H_{IP}. **b,** in-situ polar MOKE images with magnetic fields applied along [001] direction in the (110)-oriented Pt(5)/Co(1.2)/NiO(20)/MgO. The domain formation and expansion are clearly observed, indicating the existence of tilted magnetic easy axis in the whole sample. The brighter (darker) contrast indicates the upward (downward) state of perpendicular magnetization component. The scale bar is 100 μm.

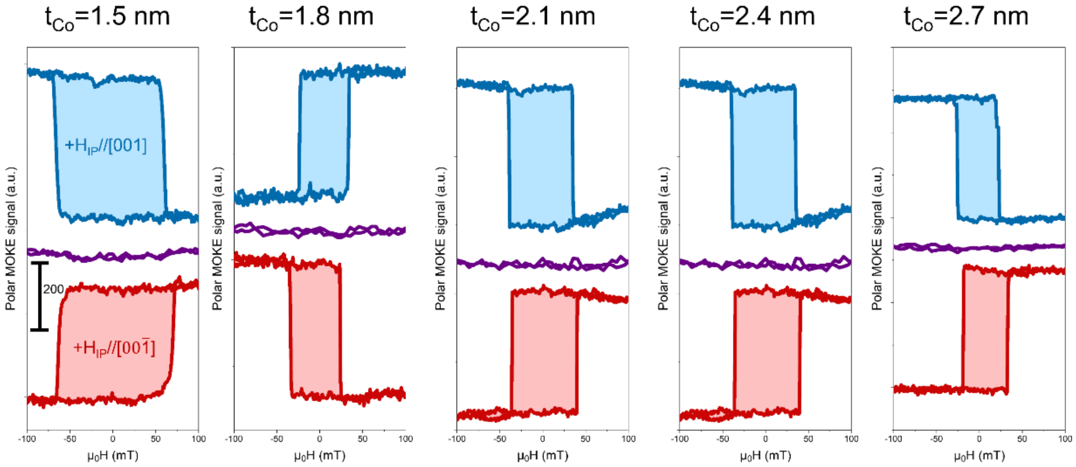

Supplementary Figure 11. The presence of tilted magnetic easy axis in heterostructures with various t_{Co}. The tilted magnetic easy axis is confirmed by switching of perpendicular magnetization component probed by polar MOKE, with sweeping in-plane magnetic field along [001] in the (110)-oriented Pt(5)/Co(t_{Co})/NiO(20)/MgO heterostructures.

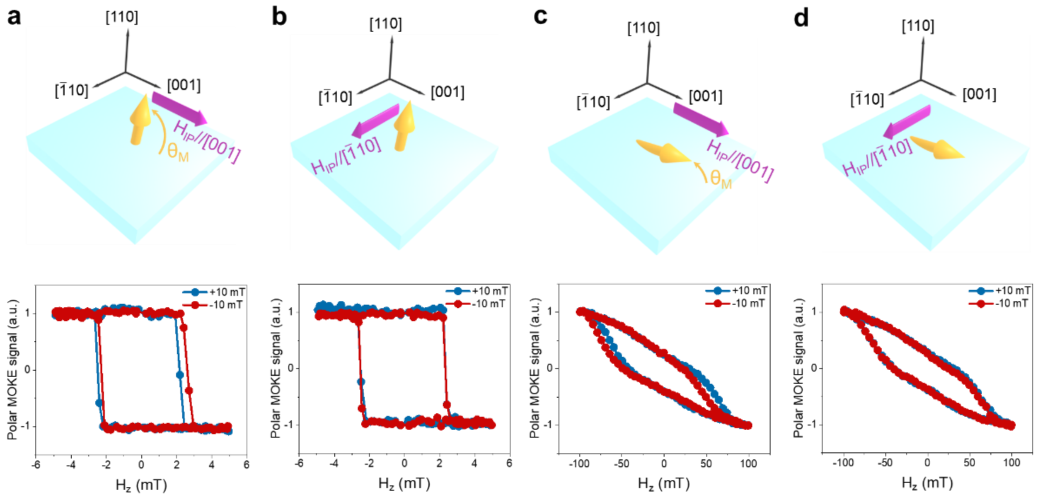

Supplementary Figure 12. The shift of polar MOKE hysteresis loop induced by in-plane bias field H_{IP} in (110)-orientated heterostructures. **a** and **b** show the polar MOKE hysteresis as sweeping out-of-plane field H_z of (110)-oriented heterostructure with θ_M=84°, when H_{IP} is applied along **a**, [001] or **b**, [$\bar{1}$10] direction. **c** and **d** show the polar MOKE hysteresis as sweeping out-of-plane field H_z of (110)-oriented heterostructure with θ_M=16°, when H_{IP} is applied along **c**, [001] or **d**, [$\bar{1}$10] direction. The upper panels show the schematic diagrams of H_{IP} and tilted magnetic easy axis. The magnitude of H_{IP} is 10 mT.

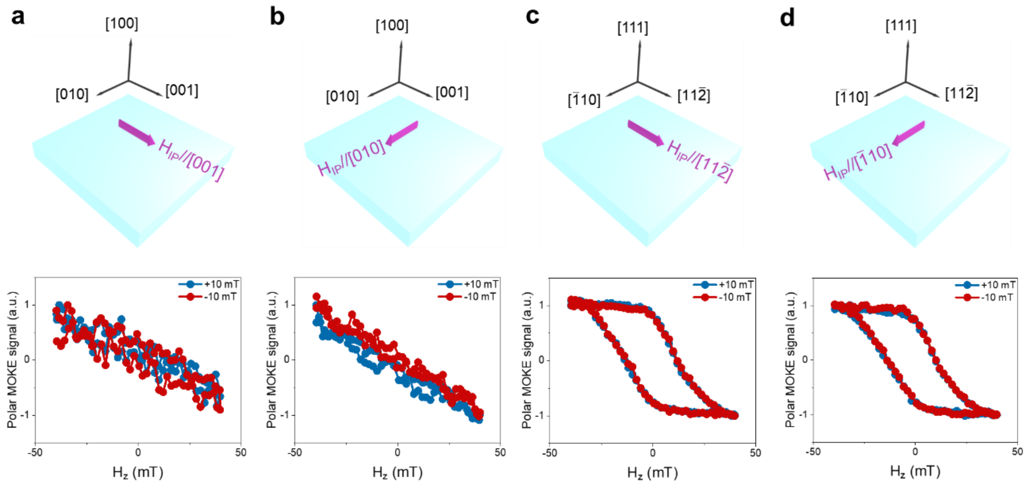

Supplementary Figure 13. The absence of shift of polar MOKE hysteresis loop with in-plane bias field H_{IP} in (100)- and (111)-oriented heterostructures. **a** and **b** show the polar MOKE hysteresis as sweeping out-of-plane field H_z of (100)-oriented heterostructure, when H_{IP} is applied along **a**, [001] or **b**, [010] direction. **c** and **d** show the polar MOKE hysteresis as sweeping out-of-plane field H_z of (111)-oriented heterostructure, when H_{IP} is applied along **c**, [11$\bar{2}$] or **d**, [$\bar{1}$10] direction. The upper panels show the schematic diagrams of H_{IP} and crystalline directions. The magnitude of H_{IP} is 10 mT.

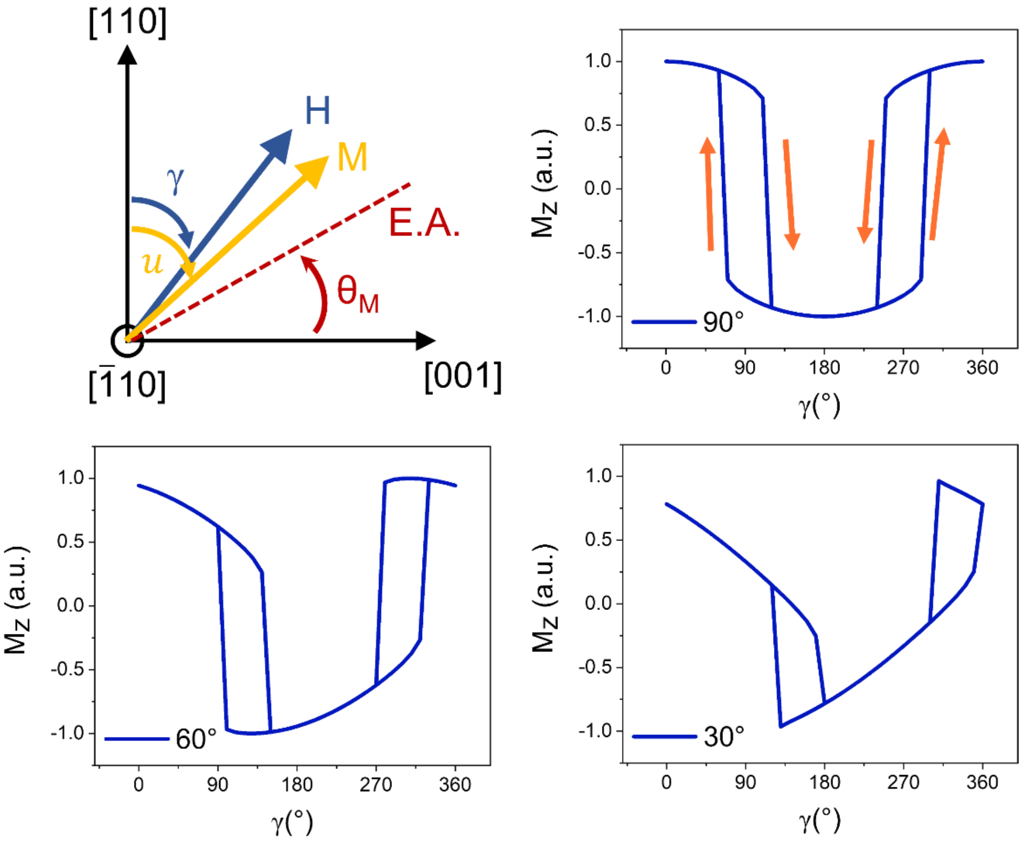

Supplementary Figure 14. Simulated polar angle γ dependence of the Hall resistivity of the samples with tilted anisotropy. The sketch of magnetic field direction (γ) and magnetic easy axis direction (θ_M) is displayed. The simulated results for θ_M = 90°, 60° and 30° are showing here, respectively. Results in Fig. 2**d** in main text is fitted with θ_M = 20°.

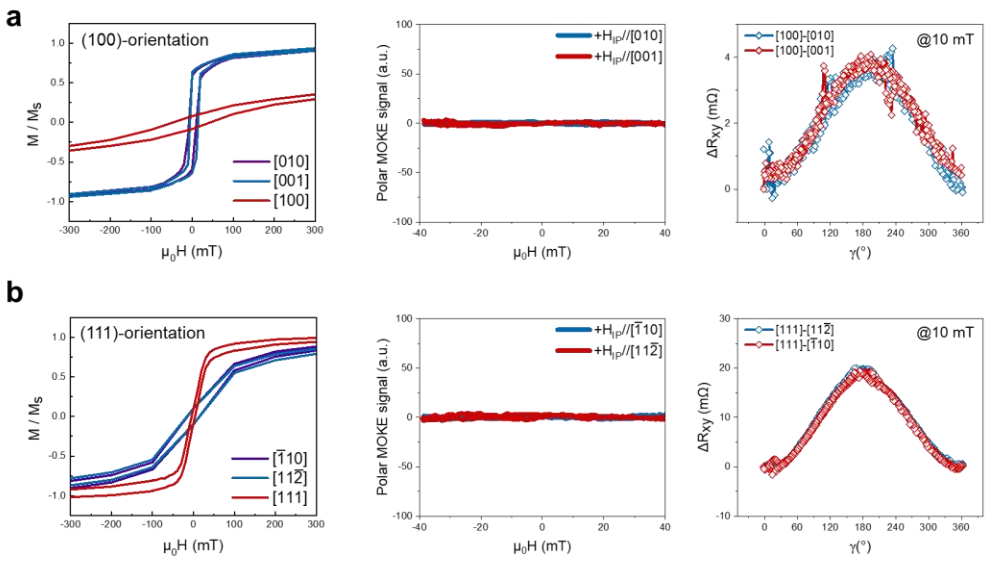

Supplementary Figure 15. The magnetic properties of Pt(5)/Co(1.2)/NiO(20)/MgO heterostructures with (100)- and (111)-orientation detected by magnetic hysteresis loops, polar MOKE signal and polar angular rotation of external magnetic field. a, the results of (100)-oriented heterostructures. **b**, the results of (111)-oriented heterostructures. The crystalline directions are all defined by substrates.

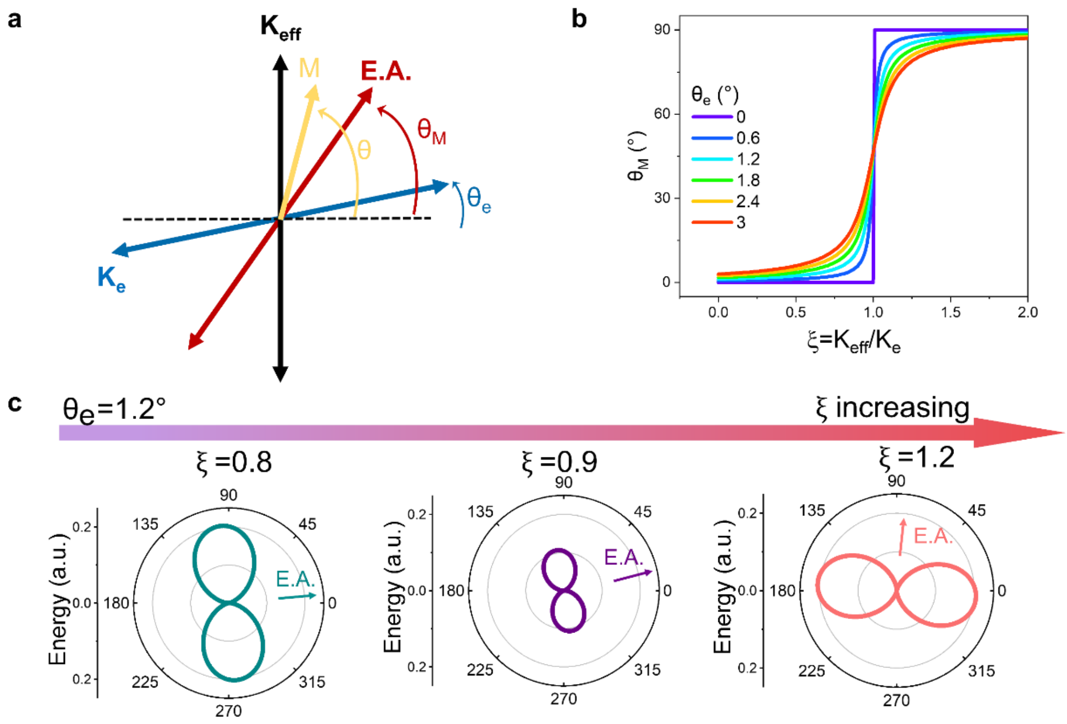

Supplementary Figure 16. Phenomenological illustration of the tilted magnetic easy axis. a, the schematic diagram of the tilted magnetic easy axis (E.A.) based on competition between K_{eff} and K_e. θ_e represents the tilting angle of lattice and θ_M represents the direction of magnetic easy axis. **b,** the dependence of θ_M on the ratio ξ with different values of θ_e. **c,** the angular dependence of anisotropy energy for $\xi = 0.8$, $\xi = 0.9$ and $\xi = 1.2$ with a fixed $\theta_e = 1.2°$. The inset arrows indicate the direction of easy axis.

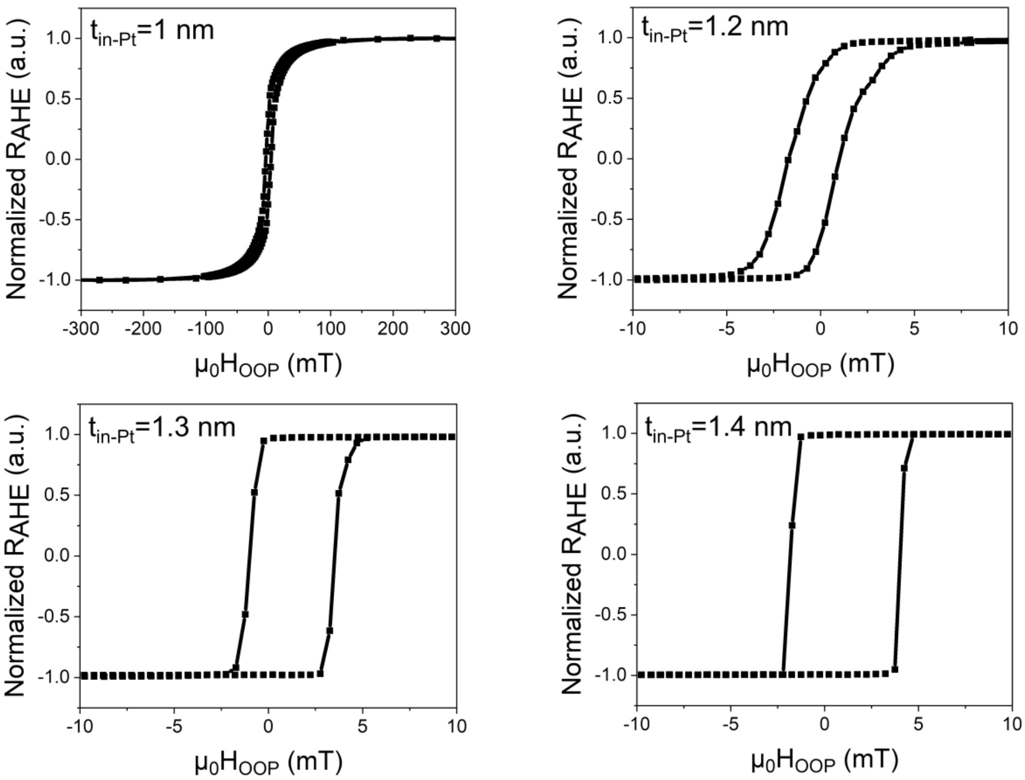

Supplementary Figure 17. **The normalized R_{xy} for heterostructures with various of $t_{in\text{-}Pt}$.** Here, samples are the (110)-oriented Pt(5)/Co(0.6)/Pt($t_{in\text{-}Pt}$)/NiO(20)/MgO heterostructures with $t_{in\text{-}Pt}$ = 1 nm, 1.2 nm, 1.3 nm, 1.4 nm, respectively.

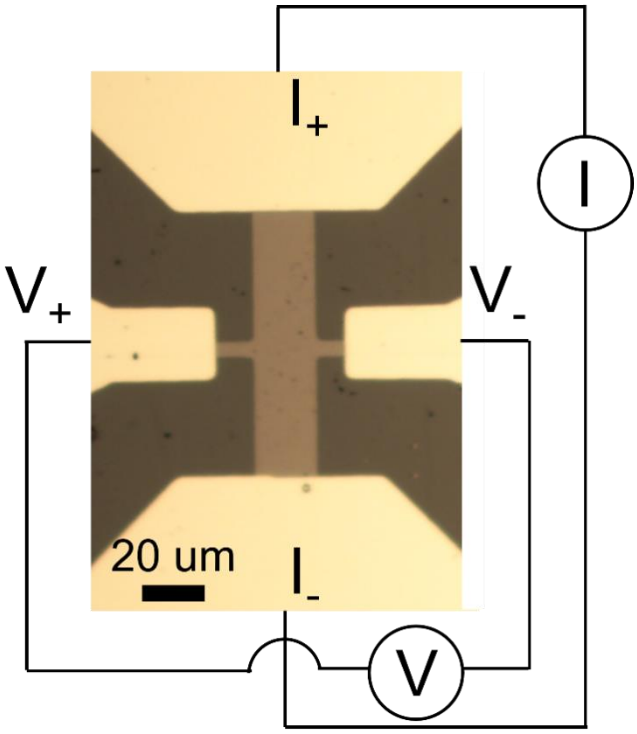

Supplementary Figure 18. The optical image of a representative Hall bar device.

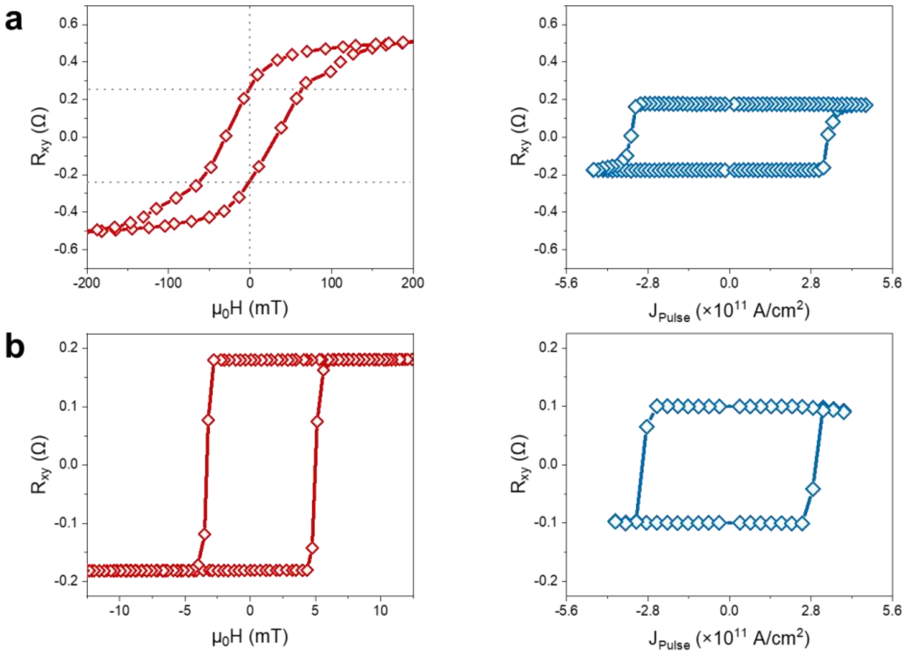

Supplementary Figure 19. **The field-free SOT switching ratio of heterostructures. a,** heterostructure Pt(5)/Co(1.2)/NiO(20)/MgO(110). The dash lines denote the remnant R_{xy} in the zero field. **b**, heterostructure Pt(5)/Co(0.6)/Pt(1.4)/NiO(20)/MgO(110). The left panel is the AHE resistance measured by external magnetic fields, and right panel shows the corresponding field-free SOT switching results. The current pulse is applied along [$\bar{1}$10]. The field-free SOT switching ratio is about 70% for heterostructure Pt(5)/Co(1.2)/NiO(20)/MgO(110), and 60% for heterostructure Pt(5)/Co(0.6)/Pt(1.4)/NiO(20)/MgO(110).

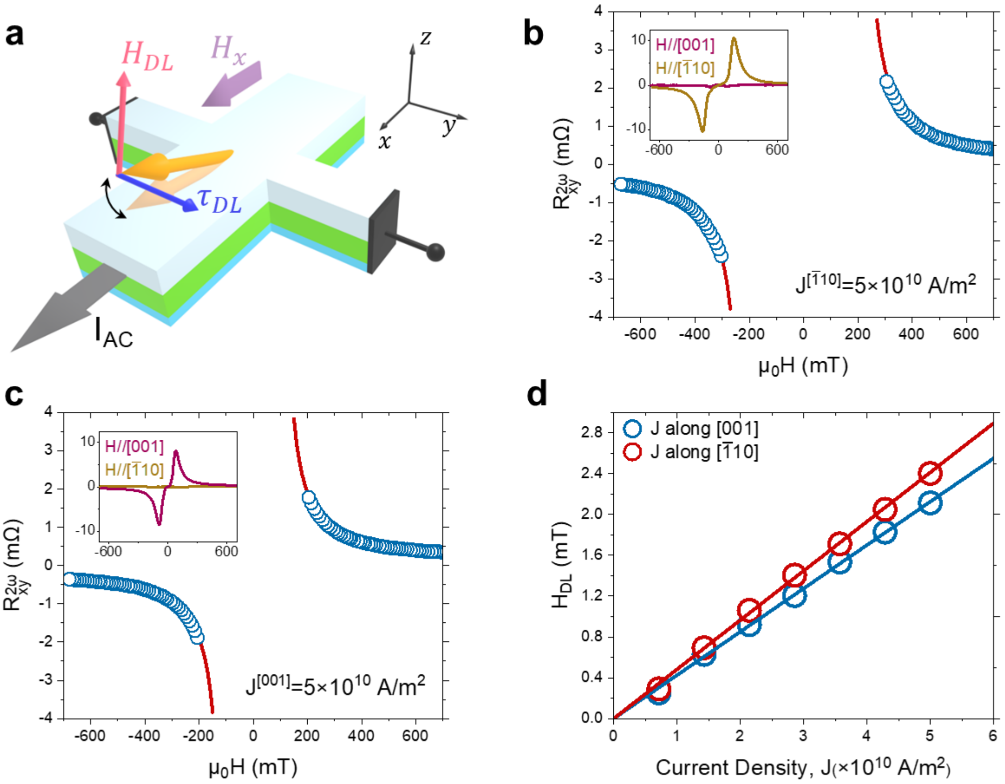

Supplementary Figure 20. SOT efficiency measured by second Harmonic Hall resistance (SHH) measurements. **a**, the schematic diagram of the SHH measurement geometry. The magnetization **M** is oscillated by H_{DL}. The yellow arrows indicate the direction of magnetization **M**. The experimental results of $R_{xy}^{2\omega}$ are shown in **b**, *ac* current along [$\bar{1}$10], and **c**, *ac* current along [001]. The blue circles represent the experimental data, and the red lines represent the fitting curve. The insets are the detected $R_{xy}^{2\omega}$ with external field H along [001] or [$\bar{1}$10], applying *ac* current with same magnitude. **d**, the effective field H_{DL} as a function of current density. The red and blue lines represent the linear fitting.

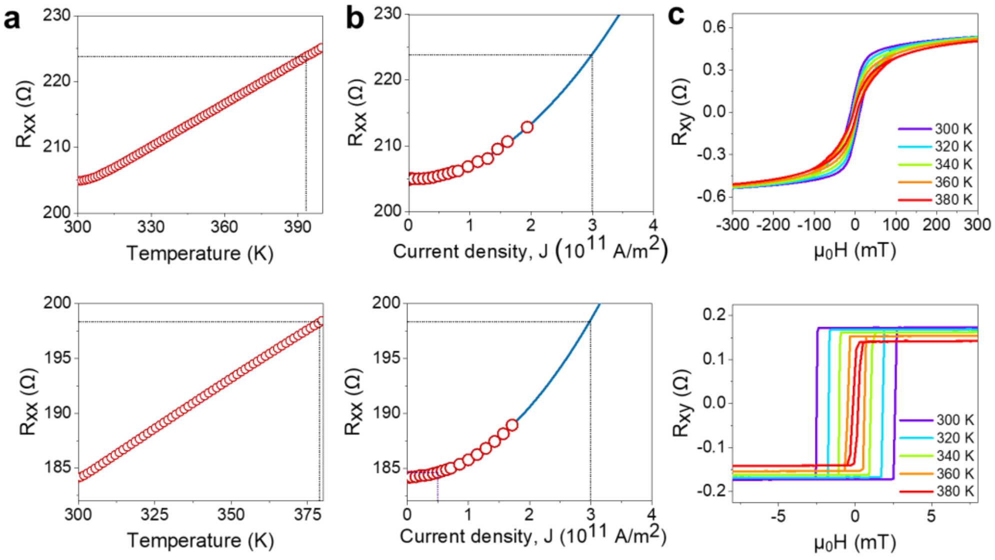

Supplementary Figure 21. **The estimated Joule heating effect induced by current pulse. a,** the temperature dependent R_{xx} with dc current of 0.1 mA. **b,** estimated temperature rising by monitoring the R_{xx} with various ac current. The red circles represent the experimental data, and the blue lines represent the parabolic fitting curve. **c,** the temperature dependent AHE resistance. Figures in upper panel correspond to heterostructure Pt(5)/Co(1.2)/NiO(20)/MgO(110) and figures in lower panel correspond to heterostructure Pt(5)/Co(0.6)/Pt(1.4)/NiO(20)/MgO(110).

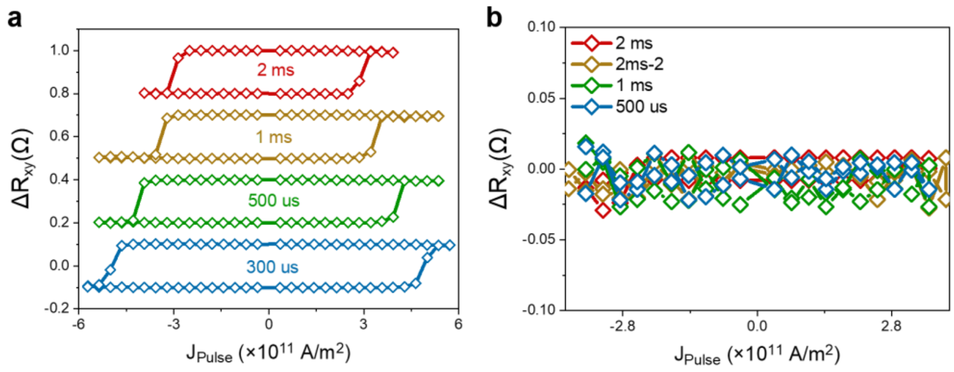

Supplementary Figure 22. The pulse-width-dependent field-free SOT switching of heterostructure with $\theta_M = 84°$. a, the change of Hall resistance when the J_{pulse} is along $[\bar{1}10]$. **b**, the change of Hall resistance when the J_{pulse} is along [001]. The data are shifted for better visualization.

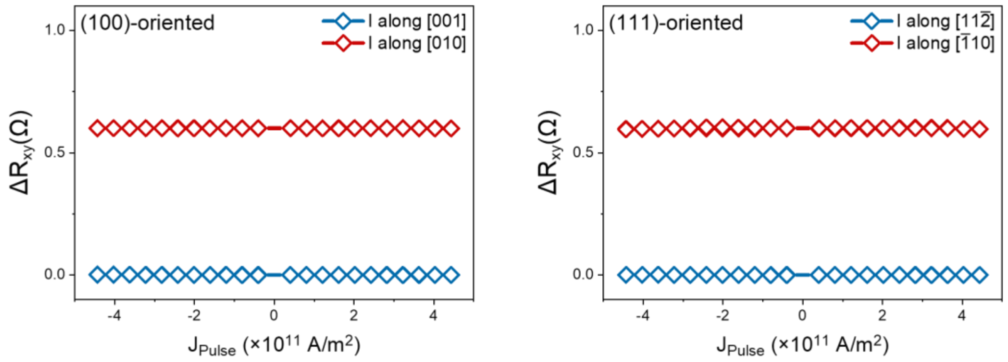

Supplementary Figure 23. The absence of field-free SOT switching of perpendicular magnetization in (100)- and (111)-oriented Pt(5)/Co(1.2)/NiO(20)/MgO heterostructures. The data are shifted for better visualization.

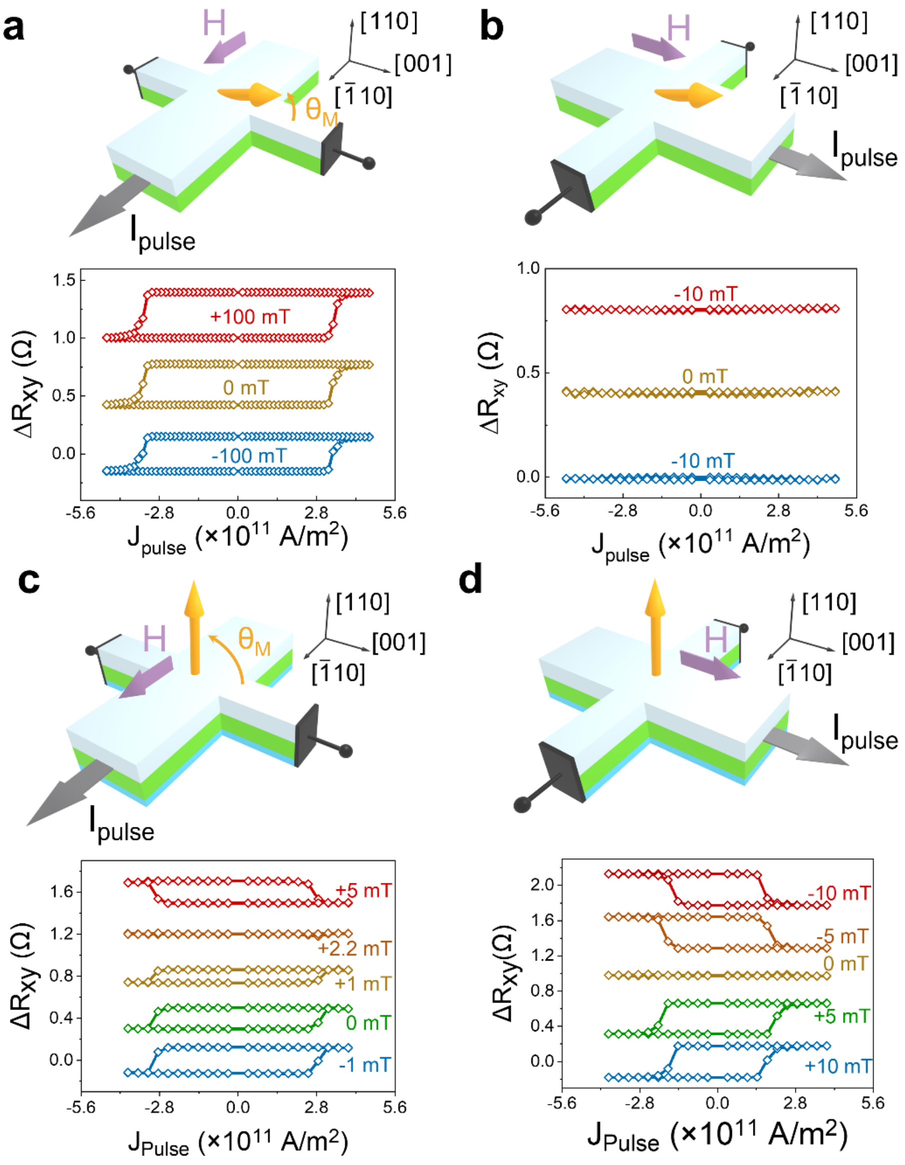

Supplementary Figure 24. The field-dependent SOT switching of perpendicular magnetization. The external magnetic field H is parallel to current pulse. **a** and **b**, the field-dependent SOT switching in Pt(5)/Co(1.2)/NiO(20)/MgO(110) (θ_M = 16°). **a**, the current pulse is applied along [$\bar{1}$10]. **b**, the current pulse is applied along [001]. **c** and **d**, the field-dependent SOT switching in Pt(5)/Co(0.6)/Pt(1.4)/NiO(20)/MgO(110) (θ_M = 84°). **c,** the current pulse is applied along [$\bar{1}$10]. **d**, the current pulse is applied along [001].

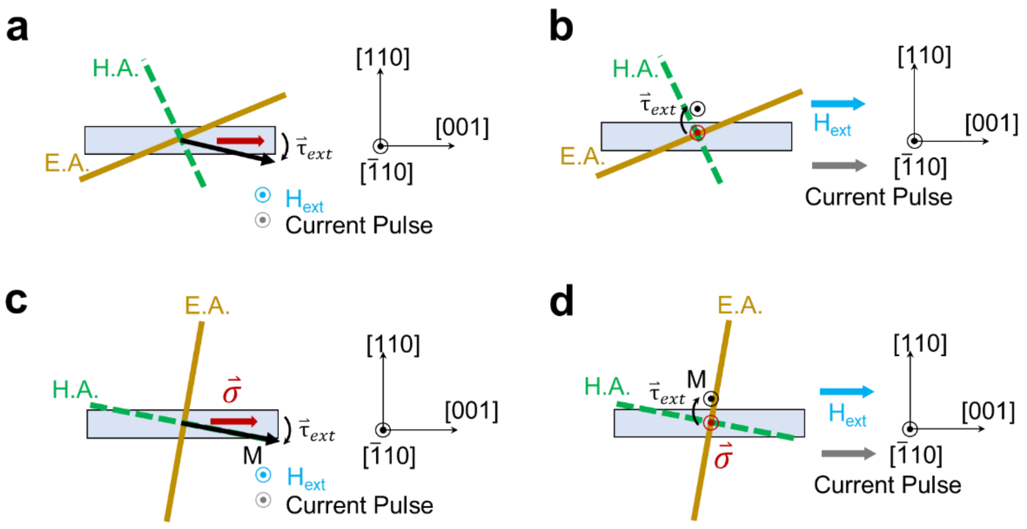

Supplementary Figure 25. The mechanism of field-dependent SOT switching of perpendicular magnetization. a-b, the effect of $\vec{\mathbf{H}}_{ext}$ in heterostructure with θ_M = 16°. **a**, the current pulse is applied along [$\bar{1}$10], the hard axis is away from sample plane and the $\vec{\boldsymbol{\tau}}_{ext}$ is insufficient to compensate SOT switching. **b**, the current pulse is applied along [001], the magnetization is strongly favored by $\vec{H}_{ext}$, thus the SOT switching is absent. **c-d**, the effect of $\vec{\mathbf{H}}_{ext}$ in heterostructure with θ_M = 84°. **c**, the current pulse is applied along [$\bar{1}$10], the $\vec{\boldsymbol{\tau}}_{ext}$ could force the magnetization align along hard axis, and the SOT switching is compensated. **d**, the current pulse is applied along [001], and the $\vec{\tau}_{ext}$ assists deterministic SOT switching. The easy axis (E.A.) is denoted by brown lines, and hard axis (H.A.) is denoted by green dash lines. Detailed discussion is included in Supplementary Note 6.

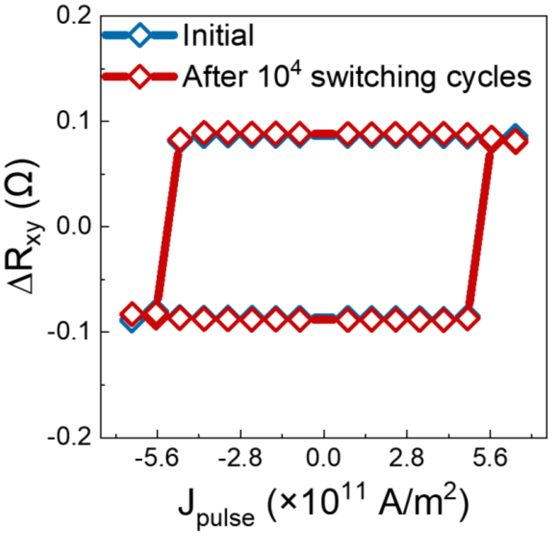

Supplementary Figure 26. Current-driven SOT switching in the initial cycle and after 1×10^4 cycles. The current (I_{pulse}) is along [$\bar{1}$10] direction with 300 μs pulse width in the (110)-oriented Pt(5)/Co(0.6)/Pt(1.4)/NiO(20)/MgO.

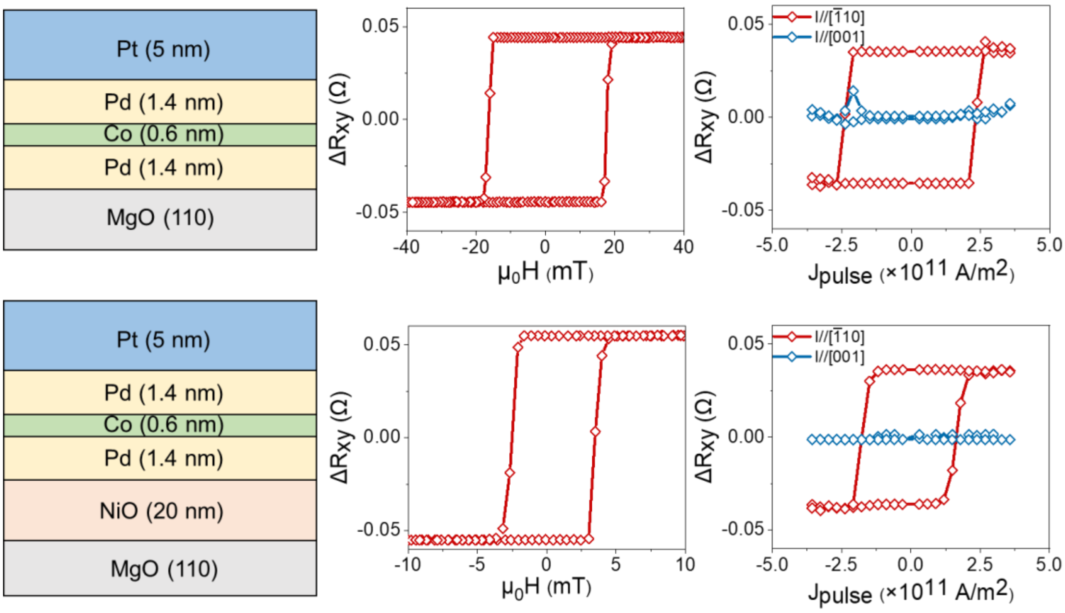

Supplementary Figure 27. Field-free SOT switching in Pd-based heterostructures. The field dependence of anomalous Hall resistance and field-free SOT switching when current pulse is applied along [$\bar{1}$10] and [001] directions in (110)-oriented Pt(5 nm)/Pd(1.4 nm)/Co(0.6 nm)/Pd(1.4 nm)/MgO and Pt(5 nm)/Pd(1.4 nm)/Co(0.6 nm)/Pd(1.4 nm)/NiO(20 nm)/MgO heterostructures.

Supplementary Table 1. The comparison with other studies based on tilted magnetic anisotropy.

Heterostructure	Strategy	FM layer Number	Fabrication	Switching Ratio ($\Delta R_{xy}/R_{xy}$)	J_c($\times 10^{11}$ A/m^2)	Endurance (cycle)	Ref
Ta/CoFeB/MgO	Device Asymmetry	1	Nano-fabrication (nano-wedged device)	~ 100%	2.5	2	15
Pt/[Gd/Co]$_9$	Interlayer Coupling	9	As-deposited	N/A	3.2	15	16
CoPt	In-plane remnant magnetization	1	As-deposited	~ 2%	7.5	16	17
Pt/Co	Dislocation	1	As-deposited	~ 70%	3	10^4	This work

Supplementary References

1. Nakamura, N. et al. Mechanism of elastic softening behavior in a superlattice. *Phys. Rev. Lett.* **99**, 035502 (2007).

2. Kan, D. et al. Tuning magnetic anisotropy by interfacially engineering the oxygen coordination environment in a transition metal oxide. *Nat. Mater.* **15**, 432-437 (2016).

3. Hillebrands, B. & Dutcher, J.R. Origin of very large in-plane anisotropies in (110)-oriented Co/Pd and Co/Pt coherent superlattices. *Phys. Rev. B* **47**, 6126 (1993).

4. Lin, C.-J. et al. Magnetic and structural properties of Co/Pt multilayers. *J. Magn. Magn. Mater.* **93**, 194-206 (1991).

5. Hillebrands, B. et al. Suppression of the magnetocrystalline bulk anisotropy in thin epitaxial Co(110) films on Cu(110). *Phys. Rev. B* **53**, R10548 (1996).

6. Johnson, M.T. et al. Magnetic anisotropy in metallic multilayers. *Rep. Prog. Phys.* **59**, 1409 (1996).

7. Wu, H. et al. Room-temperature spin-orbit torque from topological surface states. *Phys. Rev. Lett.* **123**, 207205 (2019).

8. Z. Zheng. et al. Field-free spin-orbit torque-induced switching of perpendicular magnetization in a ferrimagnetic layer with a vertical composition gradient. *Nat. Commun.* **12**, 1-9 (2021).

9. Pai, C. F. et al. Determination of spin torque efficiencies in heterostructures with perpendicular magnetic anisotropy. *Phys. Rev. B* **93**, 144409 (2016).

10. Fan, W. et al. Asymmetric spin-orbit-torque-induced magnetization switching with a noncollinear in-plane assisting magnetic field. *Phys. Rev. Appl.* **11**, 034018 (2019).

11. Liu, L. et al. Current-Induced Switching of Perpendicularly Magnetized Magnetic Layers Using Spin Torque from the Spin Hall Effect. *Phys. Rev. Lett.* **109**, 096602 (2012).

12. Liu, L. et al. Current-induced magnetization switching in all-oxide heterostructures. *Nat. Nanotechnol.* **14**, 939-944 (2019).

13. Fukami, S. et al. A spin–orbit torque switching scheme with collinear magnetic easy axis and current configuration. *Nat. Nanotechnol.* **11**, 621-625 (2016).

14. Miron, I. M. et al. Perpendicular switching of a single ferromagnetic layer induced by in-plane current injection. *Nature* **476**, 189-193 (2011).

15. You, L. et al. Switching of perpendicularly polarized nanomagnets with spin orbit torque without an external magnetic field by engineering a tilted anisotropy. *Proc. Natl. Acad. Sci. U. S. A.* **112**, 10310-10315 (2015).

16. Kim, H. J. et al. Field-Free Switching of Magnetization by Tilting the Perpendicular Magnetic Anisotropy of Gd/Co Multilayers. *Adv. Funct. Mater.* **32**, 2112561 (2022).

17. Li, Z. et al. Field-Free Magnetization Switching Induced by Bulk Spin–Orbit Torque in a (111)-Oriented CoPt Single Layer with In-Plane Remanent Magnetization. *ACS Appl. Electron. Mater.* **4**, 4033-4041 (2022).
